# Supplementary material for: Highly efficient and selective extraction of gold by reduced graphene oxide
Source: Nat Commun. 2022 Aug 2;13:4472. doi: 10.1038/s41467-022-32204-4 (PMC9345893; doi:10.1038/s41467-022-32204-4)
Supplement: Supplementary file 1 — Supplementary Information [file 41467_2022_32204_MOESM1_ESM.pdf]

Supplementary Information

Highly Efficient and Selective Extraction of Gold by Reduced Graphene Oxide

Fei Li[#1], Jiuyi Zhu[#1], Pengzhan Sun[2], Mingrui Zhang[1], Zhenqing Li[1], Dingxin Xu[1], Xinyu Gong[1], Xiaolong Zou[1], A. K. Geim[1,2]*, Yang Su[1,]*, Hui-Ming Cheng[3,4,]*

[1]Tsinghua-Berkeley Shenzhen Institute & Institute of Materials Research, Tsinghua Shenzhen International Graduate School, Tsinghua University, Shenzhen 518055, P. R. China

[2]School of Physics & Astronomy, University of Manchester, Manchester M13 9PL, United Kingdom

[3] Faculty of Materials Science and Engineering / Institute of Technology for Carbon Neutrality, Shenzhen Institute of Advanced Technology, Chinese Academy of Sciences, Shenzhen 518055, P. R. China

[4]Shenyang National Laboratory for Materials Sciences, Institute of Metal Research, Chinese Academy of Sciences, Shenyang 110016, P. R. China

[#]These authors contributed equally to this work.

*Corresponding authors: Andre.K.Geim@manchester.ac.uk (A.K.Geim); Su.yang@sz.tsinghua.edu.cn (Y. Su); Cheng@imr.ac.cn (H.-M. Cheng)

Supplementary Note 1. Comparing rGO's performance with other gold adsorbents

Supplementary Fig. 1 compares our rGO suspensions with gold adsorbents reported in the literature[1-21]. A large amount of published data is summarized in this figure, which clearly shows a trade-off between the extraction capacity and gold concentration *C* in solutions.

To highlight the superior extraction capacity of rGO, we compared rGO's extraction capacity with adsorbents that showed high extraction capacity reported elsewhere. The molybdenum disulfide modified carbon nanotubes (CNT-MoS_2), thiourea-modified porous aromatic framework (PAF-1-thiourea), porous porphyrin polymer (COP-180), and amyloid-like protein membrane (PTL membrane) showed a maximum capacity of 2495 mg/g to 1000 ppm Au ion, 2629 mg/g to 500 ppm Au ion, 1620 mg/g to 3000 ppm Au ion,1034 mg/g to 984.8 ppm Au ion, respectively. However, their capacity decreased dramatically to low concentration. For example, the extraction capacities to the lowest gold concentration studied in these reports are 1000 mg/g to 100 ppm Au ion (CNT-MoS_2), 250 mg/g to 20 ppm Au ion (PAF-1-thiourea), 100 mg/g to 20 ppm Au ion (COP-180) and 500 mg/g to 196.9 ppm Au ion (PTL membrane), respectively. In comparison, rGO showed a significantly higher capacity as shown in Supplementary Fig. 1a and Supplementary Fig. 1b. Moreover, this exceptional performance of rGO suspensions extends into the ppb and sub-ppb range where no other adsorbent was so far reported to exhibit any discernible extraction of gold (Supplementary Fig. 1c).

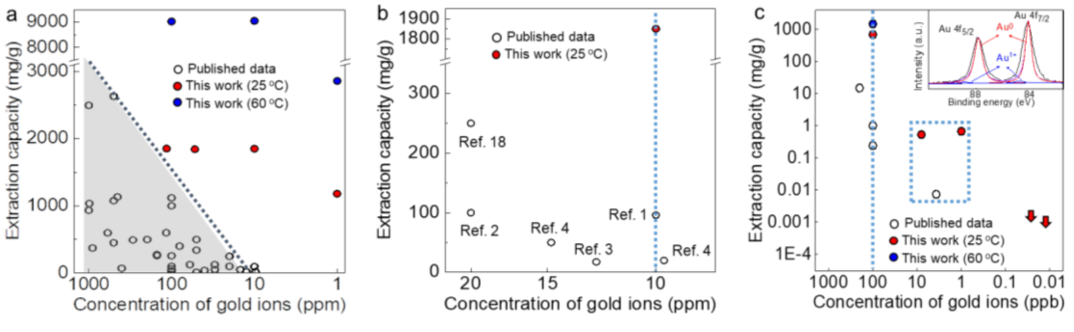

Supplementary Fig. 1| Comparison of gold extraction performance by rGO with other adsorbents. (**a**) Extraction capacity as a function of gold concentration above 1 ppm. Open symbols are the data taken from Supplementary Refs. 1-21 and listed in Supplementary Table 1. (**b**) Extraction capacity as a function of gold concentration range from 10 to 20 ppm. (**c**) Same for concentrations below 1 ppm. The red arrows show the estimated range of extraction capacity at 10 ppt and 20 ppt, we estimate ~100% extraction efficiency based on X-ray photoelectron spectroscopy (XPS) analysis. Note the logarithmic scale for the y-axis. Inset is 4*f* XPS spectrum of gold extracted by rGO from 1 ppb $[AuCl_4]^-$ measured by XPS. The open symbols are the data taken from Supplementary Refs. 1 and 22-23. All the data compared in Supplementary Fig. 1a was listed in

Supplementary Table 1.

For determination of extraction efficiency and capacity to 1 ppb, 20 ppt and 10 ppt gold solution, 0.3 mg rGO was added in 200 mL of gold solution with above concentrations. For 1 ppb gold solution, we obtained ~100 % extraction efficiency measured by inductively coupled plasma mass spectrometry (ICP-MS), which translated into an extraction capacity of ~0.7 mg/g, and similar to higher concentration, XPS analysis showed extracted gold was predominately Au^0 (Supplementary Fig. 1c inset). For rGO's adsorption to 20 ppt and 10 ppt gold solution, as ICP-MS approaches its detection limit, the rGO nanosheets after extraction were collected by centrifugation and drop-casted on a Si wafer substrate for XPS analysis. The gold contents that measured at different areas (at least 5 areas) varied from 0.01-0.18 at%. Thus, as a semi-quantitative method, XPS clearly validates significant gold extraction by rGO at ppt level.

Supplementary Table 1| Extraction capacities of various gold adsorbents. Note that all of the gold complex ion form in different references was the $[AuCl_4]^-$ or Au^{3+}, except for the $Au(S_2O_3)_2^{3-}$ in the literature 4 and 20.

gold concentration (ppm)	Types of gold ion	Gold adsorbents	Extraction capacity (mg/g)	Reference
10	$[AuCl_4]^-$	Fe-BTC/PpPDA	96	1
1000		Fe-BTC/PpPDA	934	1
20		COP-180	100	2
3000		COP-180	1620	2
12.5		TDAC	17.5	3
100		TDAC	30	3
14.8	$Au(S_2O_3)_2^{3-}$	MoS_2/CS aerogel	50	4
116		MoS_2/CS aerogel	600	4
9.7		MoS_2/CS aerogel	20	4
30	$[AuCl_4]^-$	COP-224	50	5
150		UiO-66-TA	260	6
900		UiO-66-TA	372	6
80		CSGO5	400	7
500		CSGO5	1076	7
591	Au^{3+}	$CaCu_6[(S,S)\text{-}methox]_3(OH)_2(H_2O)$	598	8

gold concentration (ppm)	Types of gold ion	Gold adsorbents	Extraction capacity (mg/g)	Reference
100	$[AuCl_4]^-$	UiO-66	60	9
100		UiO-66-NH_2	100	9
150		UiO-66-TU	275	10
30		SH-MCM-41	125	11
100		barley straw carbon	256	12
293		barley straw carbon	492	12
50		Fe_3O_4@DMSA	340	13
50		L-lysine modified, crosslinked chitosan resin	13	14
400		L-lysine modified, crosslinked chitosan resin	70.34	14
40		cross-linked lignocatechol	40	15
60	Au^{3+}	modified wheat straw	125	16
500		modified wheat straw	450	16
1000	$[AuCl_4]^-$	CNT-MoS_2	2495	17
100		CNT-MoS_2	1000	17
500		PAF-1-thiourea	2629	18
20		PAF-1-thiourea	250	18
984.8	Au^{3+}	PTL membrane	1034	19
196.9		PTL membrane	500	19
100	$Au(S_2O_3)_2^{3-}$	MoS_2/ZnS	1120	20
50		MoS_2/ZnS	500	20
450	Au^{3+}	MoS_2	1133	21
100	$[AuCl_4]^-$	rGO nanosheets at 60 ^oC	9034	this work
10			9059	
1			2858	
0.1			1480	
100		rGO nanosheets at 25 ^oC	1880	
10			1850	
1			1180	
0.1			690	

Supplementary Fig. 2 showed the change of gold extraction capacity with pH at a temperature (T) of 25 ^oC (Supplementary Fig. 2a) and 60 ^oC (Supplementary Fig. 2b). At T=25 ^oC, rGO had

extraction capacities of 700 mg/g and 340 mg/g to 10 ppm gold at pH=2 and 11 respectively. The extraction capacity can be further increased by increasing *T.* At 60 °C, rGO had a capacity of 3200 mg/g at pH=2, even at pH=0 (10 ppm Au solution containing ~2 M H^+), we still observed a capacity of 586 mg/g. At pH=11, the extraction capacity of rGO to 10 ppm gold was 1565 mg/g. In contrast, as shown in Supplementary Fig. 1 and Supplementary table 1, most adsorbents showed an extraction capacity <300 mg/g at 10 ppm, suggesting superior gold extraction performance of rGO even in strong acidic and basic solutions.

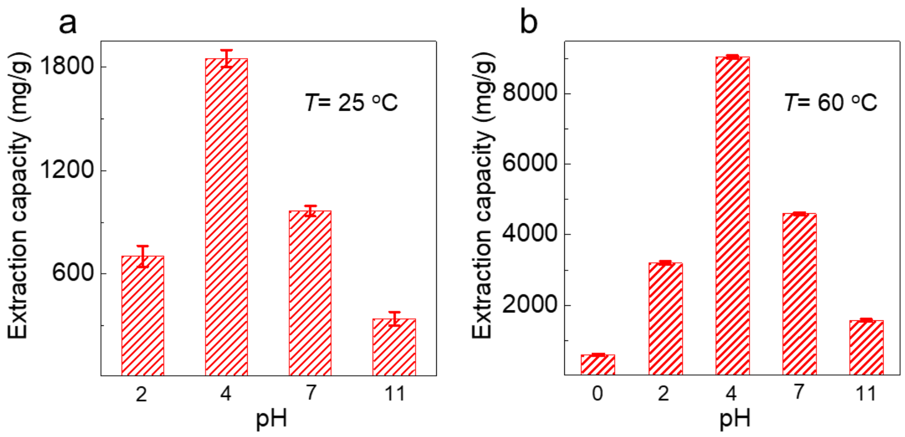

Supplementary Fig. 2| The extraction capacity measured for 10 ppm solutions at different pH. The weight ratio of gold ion and rGO was 2:1. The extraction temperature was (a) 25 °C and (b) 60 °C. All the error bars in this figure represent the standard deviation.

To study the gold adsorption isotherms of rGO, batch adsorption experiments were conducted. 2 mL rGO suspension (0.5 mg/mL) was added to 198 mL gold solution with initial concentrations of 1, 5, 10, 20, 50 and 100 ppm, respectively. After 24 hr adsorption, the extraction capacity was measured and plotted versus the equilibrium gold concentration (Supplementary Fig. 3a). At an equilibrium concentration of 0.21 ppm (10 ppm as starting concentration), the adsorption capacity reached 1850 mg/g, confirming ultrahigh gold extraction capacity. By normalizing the extraction capacity at different time, it became more evident to support the fast adsorption kinetics. Within 10 minutes, its extraction capacity was 1012 mg/g and extraction efficiency reached 54.7%, and reached 100% after 24 hr (Supplementary Fig. 3b).

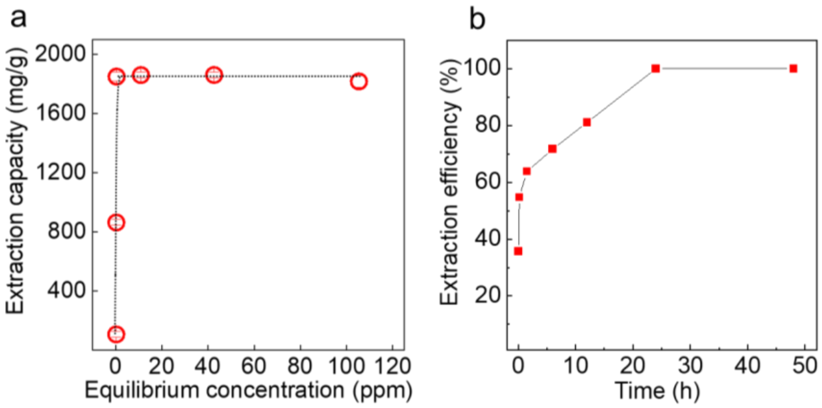

Supplementary Fig. 3| Gold adsorption isotherms and kinetics at pH=4. The extraction temperature was 25 ºC. (a) Gold adsorption isotherms. (b) Gold adsorption kinetics normalized with full capacity at pH=4. The weight ratio of gold ion and rGO was 2:1. All the error bars in this figure represent the standard deviation.

Supplementary Note 2. Characterization of graphene oxide and rGO before and after Au extraction

To characterize our graphene-based materials, we used X-ray diffraction (XRD) combined with other analytical techniques, including XPS and Fourier transform infrared (FTIR) spectroscopy (Nicolet iS50). The XRD patterns of GO, rGO before and rGO after Au extraction (denoted below as rGO-Au, 10 ppm $[AuCl_4]^-$ was used) are shown in Supplementary Fig. 4a. The XRD confirmed that GO was reduced by ascorbic acid. Indeed, the ~10° peak known for GO disappeared whereas another peak characteristic of rGO emerged at ~23°. The gold on rGO-Au was found to be metallic as determined from its XRD peaks (XRD peak was fitted according to JCPDS data, No. 04-0784).

The FTIR spectra of GO, rGO, and rGO-Au are shown in Supplementary Fig. 4b. The peaks at 3431, 1725 and 1396 cm^{-1} are assigned to the O-H stretching vibrations, C=O stretching and O-H flexural vibration modes of carboxylic groups, respectively[24]. In rGO and rGO-Au samples, the intensities of peaks at 3431 cm^{-1} and 1725 cm^{-1} decreased notably compared to the GO, indicating the removal of functional groups. The peak at 1396 cm^{-1} is still visible in both rGO spectra, yielding the presence of residual oxygen-containing functional groups that contribute to the stability of our rGO dispersions. The FTIR spectrum of rGO-Au exhibited no notable changes as compared to rGO, which is consistent with our interpretation that gold ions are adsorbed mostly onto graphitic regions of rGO (see the main text).

To validate gold ion has been reduced to metallic gold during extraction, thermogravimetric (TG) and differential scanning calorimetry (DSC) were used to analyse rGO sample after 24 hours

extraction (rGO-Au-24 h). Supplementary Fig. 4c shows the TG and DSC curves of rGO and $KAuCl_4$.The weight losses of rGO and $KAuCl_4$ during heating were ascribed to the decomposition of rGO and $[AuCl_4]^-$ →Au^0, respectively. For $KAuCl_4$, such transformation led to an endothermic peak at ~330 °C, which is in agreement with the previous report[25]. In contrast, this peak was absent for rGO-Au-24 h (Supplementary Fig. 4d), suggesting the extracted gold in rGO-Au-24 h was mainly Au^0 other than $[AuCl_4]^-$. Furthermore, after 700 °C calcination, the rGO-Au-24 h remained ~69 wt% of its original weight (Supplementary Fig. 4d), giving an extraction capacity of ~2100 mg/g (considering 3.6 wt% remained ash for pristine rGO (Supplementary Fig. 4c), in good agreement with the extraction capacity measured by ICP-MS (Fig. 1b in the main text).

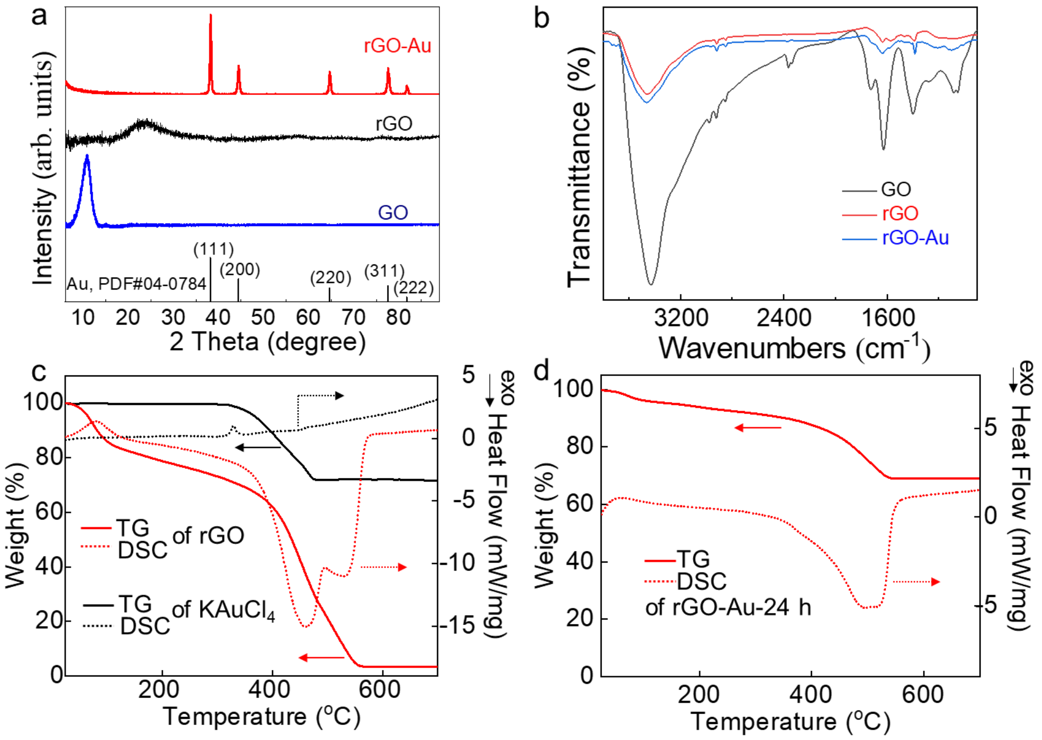

Supplementary Fig. 4| Characterization of GO, rGO, and rGO-Au. (**a**) Their XRD patterns and (**b**) FTIR analyses. (**c**) TG and differential scanning calorimetry (DSC) curves of $KAuCl_4$, rGO, and (d) rGO-Au after 24 h extraction (rGO-Au-24 h) measured in air.

The Raman spectra in Supplementary Fig. 5a show that the I_D/I_G ratios of rGO and rGO-Au are 1.06 and 0.94, respectively. The defect density in the carbon materials, especially graphene materials is characterized by L_D, the distance between two neighboring defects. Obviously, a higher L_D suggests a less defective carbon material[26]. L_D could be determined from I_D/I_G, and generally does not monotonically change with I_D/I_G. At a small L_D, an increase in I_D/I_G suggests an increased L_D. After reaching a maximum, L_D further increases with decreased I_D/I_G. GO and rGO are reported with a

small L_D that increases with an increased I_D/I_G. GO and rGO are reported with a small L_D that decreases with a decreased I_D/I_G, therefore, the observed decrement of I_D/I_G after gold extraction suggested a more defective state of rGO, supporting the electron donation from rGO to gold. In addition, G band of rGO after gold extraction showed a blueshift from 1602 to 1606 cm^{-1}, confirming a p-doping and electron transfer from rGO to gold[27].

Supplementary Fig. 6 are the Raman map of G and D bands of rGO before and after the gold extraction at 25 °C and 60 °C respectively. We found that, firstly, because of the existence of gold on the rGO surface, the surface enhanced Raman scattering (SERS) effect emerged. Specifically, I_D peak showed an intensity range from 300-1400 for rGO, increased to 1500-2600 after gold extraction, and I_G peak increased from 300-1200 to 1400-2200. Secondly, such mapping allowed us to summarize the change of I_D/I_G before and after gold extraction. In good agreement with Supplementary Fig. 6a, I_D/I_G decreased from a range of 1.00-1.15 to 0.93-1.02 after extraction, suggesting a more defective rGO after gold extraction, because of the electron donation.

Supplementary Fig. 5b shows the UV-Vis spectra of GO, rGO, and rGO-Au. Compared to GO, the peak at 227 nm corresponding to $\pi \rightarrow \pi^*$ transitions of aromatic C–C bonds shifts to 263 nm after reduction, indicating the restoration of the electronic conjugation within the graphene sheets[28]. After mixing the rGO with $[AuCl_4]^-$, i.e. sample rGO-Au, we observed a blueshift of rGO characteristic absorption peak from 263 nm to 232 nm after 24 hr extraction, indicating electron transfer from the graphitic area to $[AuCl_4]^-$ and reducing $[AuCl_4]^-$ to Au^0, the adsorption peak of Au^0 located at 555 nm, which is typical for gold nanoparticles.

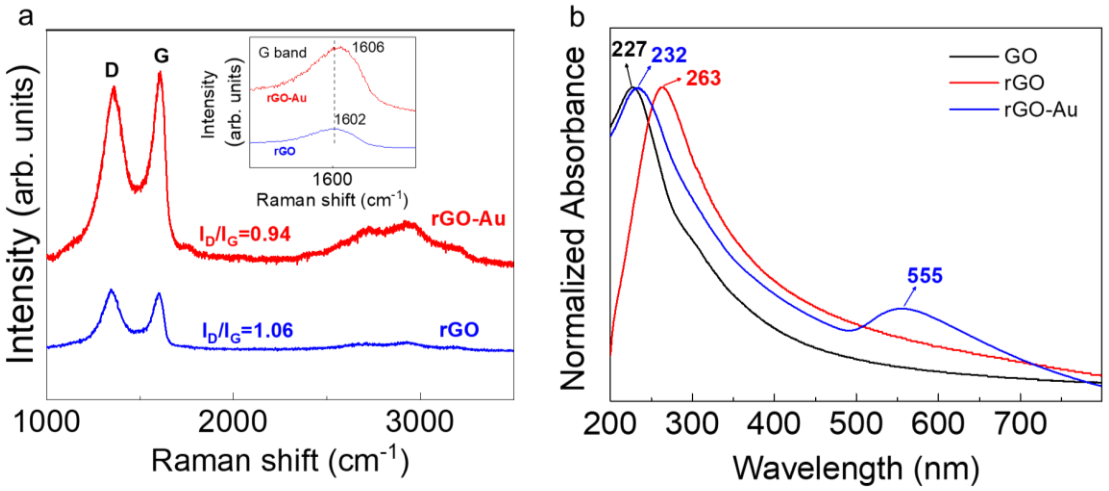

Supplementary Fig. 5| Evolution of rGO during the gold extraction process. (a) Raman spectra

of the rGO and rGO-Au. The inset shows the blueshift of G peak from 1602 to 1606 cm^{-1} after gold extraction. (b) UV-Vis spectra of GO, rGO, and rGO-Au.

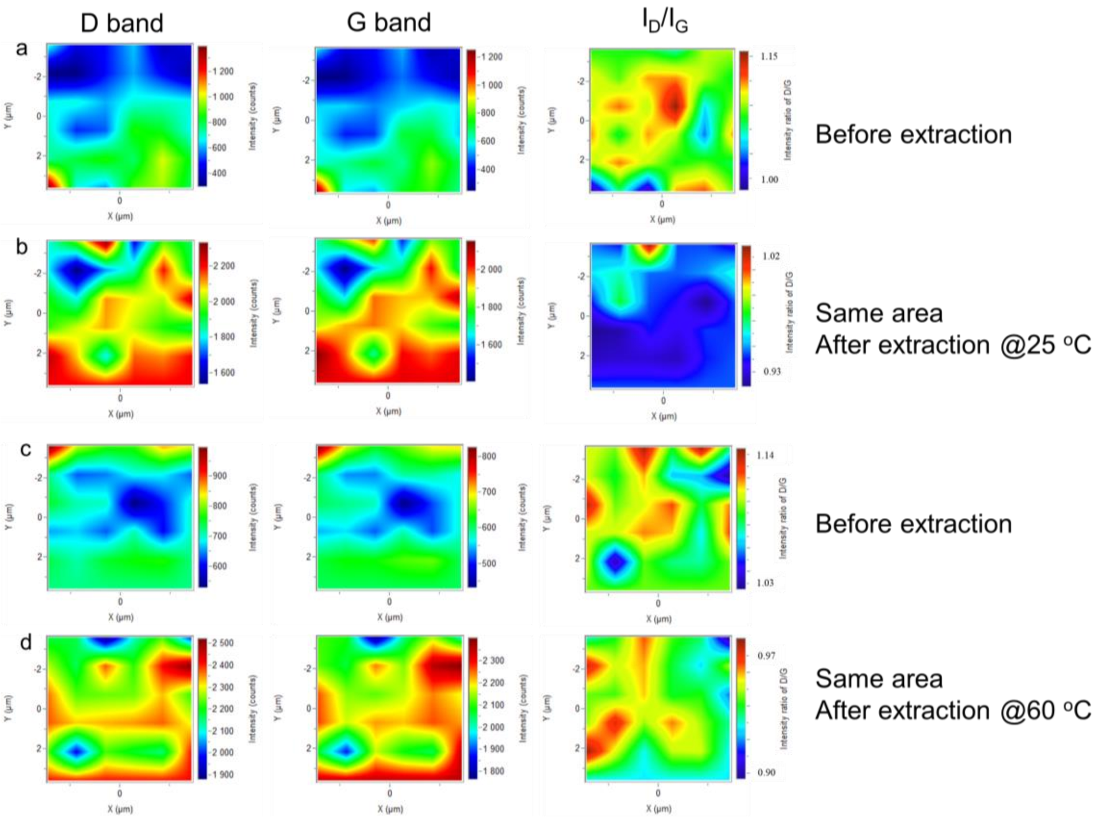

Supplementary Fig. 6| Raman map images for D, G and D/G of rGO before and after gold extraction at 25 °C and 60 °C: (a) D band, G band, and I_D/I_G before gold extraction at 25 °C. (b) D band, G band, and I_D/I_G after gold extraction at 25 °C. (c) D band, G band, and I_D/I_G before gold extraction at 60 °C. (d) D band, G band, and I_D/I_G after gold extraction at 60 °C. Note that each band was scanned in the same area of 7.2 × 7.2 μm^2.

Supplementary Note 3. Changes in rGO during gold extraction

SEM was employed to observe changes in rGO during the gold extraction process (Supplementary Fig. 7). It can be seen that, even after 2 minutes, gold nanoparticles already appeared, suggesting that the reductive adsorption mechanism kicked in. It was clear that each gold particle has an intra-particle distance between tens nanometers to a few hundred nanometers, this suggested the electron transfer needed for reductive adsorption may only require electron transfer in the sub-micrometre range, so that the interconnected graphene areas of rGO were able to provide electrons and reduce gold ion at its vicinity.

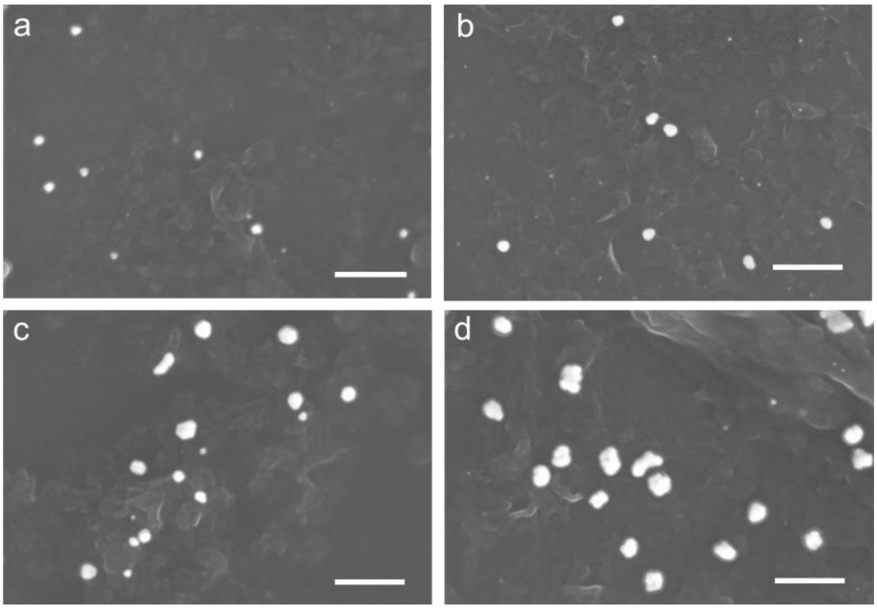

Supplementary Fig. 7| SEM images of rGO nanosheets after their exposure to a 10 ppm gold solution of $KAuCl_4$ for 2 min, 10 min, 1.5 h, and 12 h. Panels (a), (b), (c), and (d) respectively. All scale bars, 200 nm.

To check whether it is metallic gold or $KAuCl_4$ salt adsorption dominated in the early stages, we analysed rGO-Au after 10 minutes extraction (rGO-Au-10 min, 10 ppm gold solution is used) by TG and DSC analysis (Supplementary Fig. 8). Similar to rGO-Au-24 h, we did not observe the corresponding peak for $[AuCl_4]^- \rightarrow Au^0$ for rGO-Au-10 min. This is similar to the behaviour found for rGO-Au after 24 h reduction (Supplementary Fig. 4d), and confirms that the reductive adsorption mechanism takes place rapidly, at least within less than a few minutes. In addition, TG showed ~56.7 wt% for rGO-Au-10 min, which gives an extraction capacity ~1.2 g/g for 10 min gold extraction, in good agreement with the capacity measured by ICP-MS (Fig. 1c in the main text).

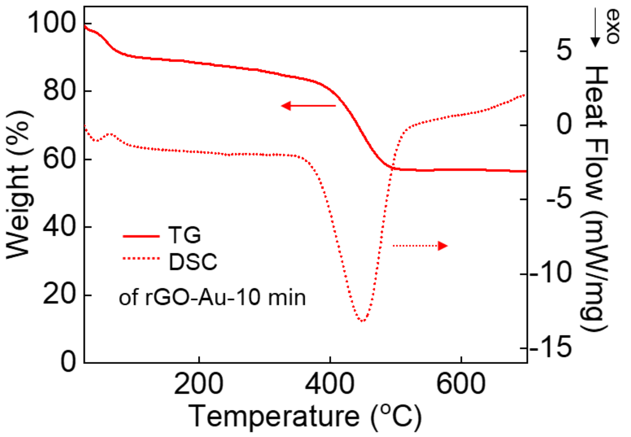

Supplementary Fig. 8| TG and DSC curves of $KAuCl_4$ and rGO-Au-10 min measured in air.

Supplementary Note 4. Gold reduction on mechanically exfoliated graphene

To gain further insight into the Au reduction mechanism, we studied the influence of graphene's thickness and morphology on its gold extraction ability. To this end, we prepared pristine graphene crystals by the standard exfoliation technique on top of an oxidized Si wafer[29]. Their thickness (the number of layers, N) was identified using optical contrast[29]. The obtained crystals were then exposed to a 10 ppm $KAuCl_4$ aqueous solution. Supplementary Fig. 9 exemplifies our observations. The SEM image shows a region covered with mono- and bi- layer graphene. After its exposure to the Au solution for 5 minutes, many areas of monolayer graphene became scrolled, warped and folded. These structural distortions were also observed in bilayer and few-layer regions (Supplementary Fig. 9) whereas multilayer graphene and graphite crystals remained flat (not shown). These pronounced changes in morphology are attributed to water permeating under exfoliated crystals, which weakened their adhesion to the SiO_2 substrate allowing the folding. We note such morphology changes did not require any presence of Au salts and occurred in both pure water and Au salt solutions before drying samples. For example, these changes in morphology of graphene crystals were observed in an optical microscope in situ, inside deionized water.

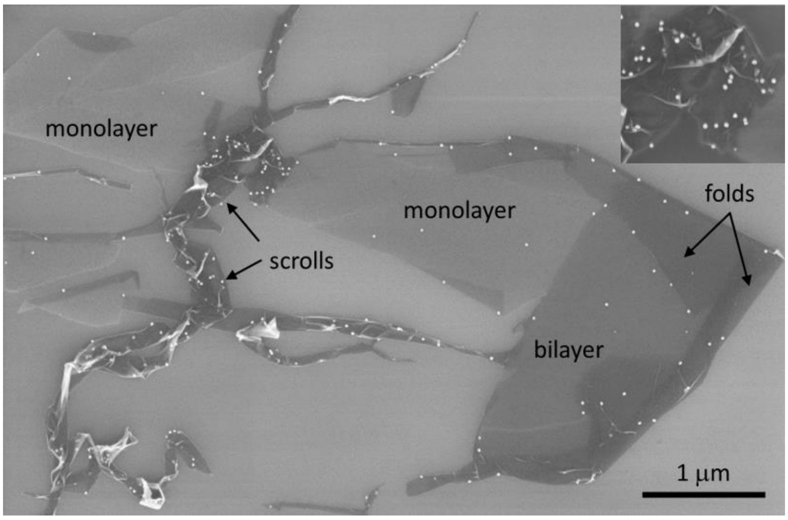

Supplementary Fig. 9| Gold extraction on flat and warped areas. Micrograph of graphene after its exposure to a 10 ppm Au ion solution for 5 minutes. Gold nanoparticles (seen as white dots and confirmed by EDS) were mostly found on top of scrolls and wrinkles, and the nanoparticles also decorated edges of folded areas. Inset: Zoom-in of the warped graphene area in the centre of the top-left quadrant. The SEM images were taken at 5 kV using electron microscope ULTRA by Zeiss.

Supplementary Fig. 10 shows that Au nanoparticles (~ 20 nm in diameter) were found mostly on top of warped and folded regions with only a few particles present in flat areas. By analysing the

resulting surface coverage using many such SEM images, we evaluated that the flat areas contained ~0.1 mg of Au per m^2 and that there was no statistically significant difference in the amount of gold deposited onto flat regions of different thicknesses (we analysed areas with N from 1 to 5 and thick graphite). The above amount translates into the extraction capacity M of ∼150 mg of Au per g of monolayer graphene and progressively less for thicker crystals ($M \propto 1/N$). It is difficult to evaluate accurately M for warped areas because of their unknown and varying thickness. However, the SEM contrast suggests that they contained graphene monolayers (and occasionally bilayers) folded only a few times. This allows us to estimate M for warped graphene as ~ 1,000 mg per g of carbon, in agreement with the extraction capacity observed for rGO for short times (Fig. 1c in the main text). In this respect, it is important to emphasize that rGO also consists of scrolled, wrinkled and folded areas rather than flat graphene.

Next, similar samples containing graphene and graphite were exposed to the same 10 ppm Au solution for 19 hr. Again, Au nanoparticles were found to heavily cover warped areas, but the coverage of flat areas was also denser allowing statistical analysis. The results are summarized in Supplementary Fig. 10 and Fig. 2e. First, Au nanoparticles became noticeably bigger for all N and occasionally could reach up to 100 nm in size. The particles also acquired irregular shapes as shown in Supplementary Fig. 10, suggesting a merger of several smaller particles. The areal Au extraction was found to be highest for monolayers, decaying with increasing N but recovering to mid values for thick graphite crystals. This behaviour is illustrated by micrographs of Supplementary Fig. 10 and quantified in Fig. 2e. We attribute the higher coverage observed for $N = 1$ to the fact that visibly flat areas of monolayer graphene were not atomically flat but followed the morphology of the oxidized Si wafer. Ripples on graphene were previously shown to be catalytically active[30]. Accordingly, the high coverage of monolayers could be due to the same effect as seen in Supplementary Fig. 9 for warped graphene. For larger N, crystals became increasingly flat, leading to fewer Au nanoparticles. It remains to be understood why graphite surfaces also contained a reasonably high Au coverage, higher than that on few-layer graphene. To this end, we note that our graphite crystals contained cleavage steps and some folded areas. This allows us to speculate that Au reduction occurred predominantly on the steps and folds (catalytically active features) and then nanoparticles migrated along the atomically flat surfaces of graphite crystals, leading to their relatively uniform coverage. Results of Supplementary Fig. 10 and Fig. 2e yield the extraction

capacity M for monolayer graphene of ~ 6,000 mg per gram of graphene, that is, ~3 times higher than for rGO in Fig. 1 of the main text. Such enhancement is perhaps not surprising because rGO nanosheets 1) contain oxidized areas (that is, not the entire surface could take part in reduction and it might contribute to the energy barrier for gold reduction as observed in Fig. 2c) and 2) tend to coagulate after initial stages of gold adsorption so that some of the graphene areas become inaccessible to gold ions.

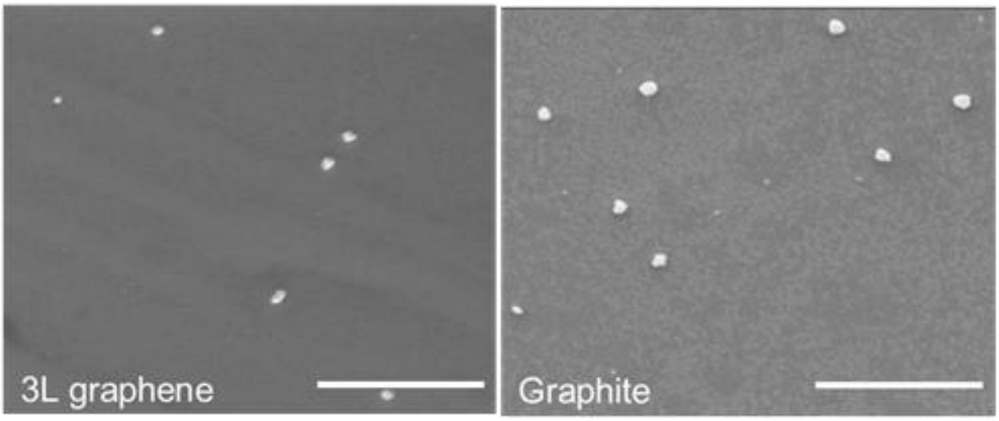

Supplementary Fig. 10| Gold extraction for graphene of different thicknesses. Micrographs illustrating typical Au coverage for flat areas of graphene with $N = 3$ and graphite. Note, N=1 and 5 are presented as insets in Fig. 2c in main text. Exposure: 19 hours in a 10 ppm Au solution. Scale bars, 1 μm for all images.

To gain the understanding on why warped area of graphene enhance gold extraction behavior, we studied adsorption energy and charge transfer between gold ion and graphene using First-principle calculation (Supplementary Fig. 11). All calculations were carried out using the Vienna Ab initio Simulation Package (VASP)[31,32] based on density functional theory (DFT)[33,34] with the Perdew-Burke-Ernzerhof functional[35]. As shown in Supplementary Fig. 11a and b, $AuCl_3$ cluster was adopted to represent the valence state of Au^{3+}, while (10, 10) carbon nanotube (CNT) was chosen as a similarity of the warped and curved surface of graphene. 6×6×1 and 1×1×5 supercells for graphene and nanotube were constructed to study the adsorption of Au^{3+}. The vacuum layers were set as at least 12 Å to avoid spurious interactions among periodic images. Zero damping DFT-D3 method[36] was applied to describe van der Waals interaction. The adsorption energy was defined as $E=E_T-E_C-E_{AuCl3}$, where E_T, E_C and E_{AuCl3} are the total energies of the adsorption system, graphene or nanotube supercell, and $AuCl_3$ cluster, respectively.

Our results show that, in contrast to flat graphene, the adsorption energy of gold ion on curved graphene surface is about 0.1 eV lower than that on graphene, indicating its preferred adsorption on

the curved graphene surface. After adsorption, the electron transfer process from graphene to gold ion drives the reduction of gold ion to Au^0. Our calculation shows that, the curved graphene has a Fermi level ~0.3 eV higher than that of flat graphene, this leads to a more significant charge transfer from curved graphene. This is further validated by Bader charge analysis, that we found that the numbers of electrons transferred from curved and flat graphene to gold ion are 0.61 and 0.47, respectively, in good agreement with observed significant gold reductive adsorption on curved surfaces.

To further determine the difference in electron transfer, we also performed gas-phase calculations on cluster models, which can take different charge states. Supplementary Fig. 11c and d show the structural models for Au-adsorbed graphene and CNT clusters, which were saturated with H atoms. The +3 charge state of the cluster models was realized by artificially setting the number of electrons, and the compensating background charge was included to ensure the convergence of electrostatic energy. After structural relaxation, the Bader charge analysis shows that the number of electrons transferred from CNT and graphene clusters to Au^{3+} was 3.03 and 2.81, respectively. Compared to graphene, CNT transferred 0.22 more electrons to Au^{3+}, which is consistent with the calculation based on $AuCl_3$ models (0.13 e). Here, we used the idealized charged systems, and a jellium background charge was added to ensure the whole system is neutral. It should be noted that, the jellium charge might introduce spurious states in the vacuum[37], which could result in the change to calculated one-electron energy and charge distribution and thus the Bader charge. Nevertheless, our DFT calculations showed a consistent trend with the experimental results. To obtain more accurate results, a self-consistent correction[37] or gas-phase simulations should be explored.

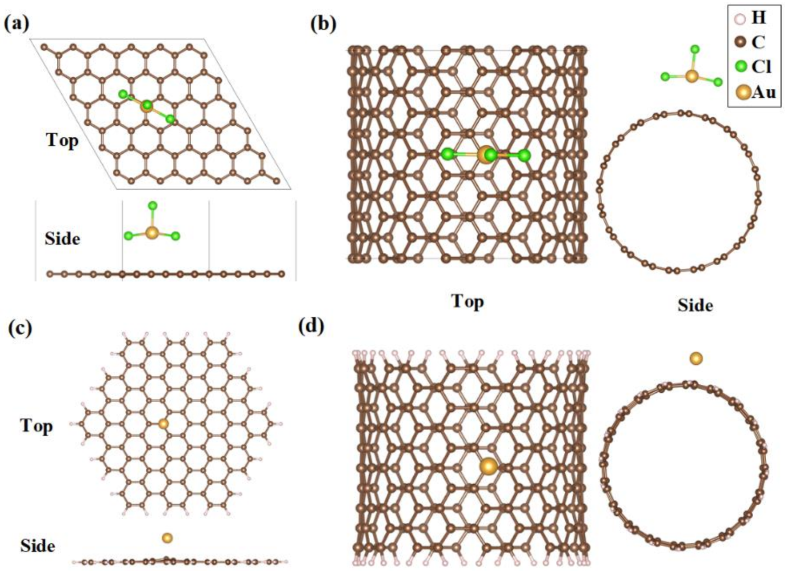

Supplementary Fig. 11| The models of gold ion adsorption on (a) graphene and (b) CNT. Gold ion is placed on top of carbon atom as it is the most stable configuration of adsorption. Top and side views of Au-adsorbed (c) graphene and (d) CNT clusters.

To summarize, our observations for exfoliated graphene suggest that both surface area and thickness are important for efficient Au extraction. The available area is obviously maximal for monolayer graphene, as in rGO's solutions and membranes used in our work. The extraction capacity is negligible for graphite (in terms of gold extracted per gram) and rapidly increases as $1/N$ with decreasing the graphite thickness N. Moreover, monolayer graphene is also beneficial for Au reduction by speeding up the process on top of uneven and warped areas, which are abundant within rGO nanosheets. In addition, our experiments on exfoliated graphene confirmed that it was pristine graphene areas that were important for Au reduction, and the minority rGO areas that remain oxidized played little role in the process.

Supplementary Note 5. Evaluation of different graphene-based adsorbents

For the rGO reduced by ascorbic acid with different time, we have used XPS, specifically, changes in C/O ratio of resulting rGO, to confirm that control of reduction time can tune the oxidized region of rGO. As shown in Supplementary Fig. 12, pristine GO showed a C/O ratio of 2.2, which has increased to 4.2, 4.7, 5.1, 5.7 for a reduction time of 10 min, 30 min, 1 h, 4 h respectively, as discussed in the previous paper, such increase of C/O ratio was strong evidence for the removal of oxidized region[24,38].

For the rGO reduced by hydrazine and hydroquinone, commercial graphene (bought from Nanjing XFNANO Materials Tech Co., Ltd.) and expanded graphite, to supplement the main text conclusion, we focused on their zeta potentials (Supplementary Fig. 12b). All the GO-based materials exhibited negative zeta potentials > |30 mV|. Such values are considered to be sufficient to provide a stable colloid[39]. In contrast, commercial graphene and expanded graphite, both had well-retained graphene areas, as confirmed by a prominent G peak and a very weak D peak from the Raman analysis (Supplementary Fig. 12c), but they either floated on or settled in the aqueous solution, failed to form a stable colloidal dispersion in water (inset of Supplementary Fig. 12b).

It is interesting to note that hydroquinone-reduced GO showed a lower zeta potential (in the absolute value), which was also accompanied by a lower extraction capacity of this rGO (Fig. 2g), as compared to the characteristics observed for ascorbic acid- and hydrazine-reduced GO. These observations indicate that the colloidal stability of rGO influences the accessibility of graphene areas for Au ions.

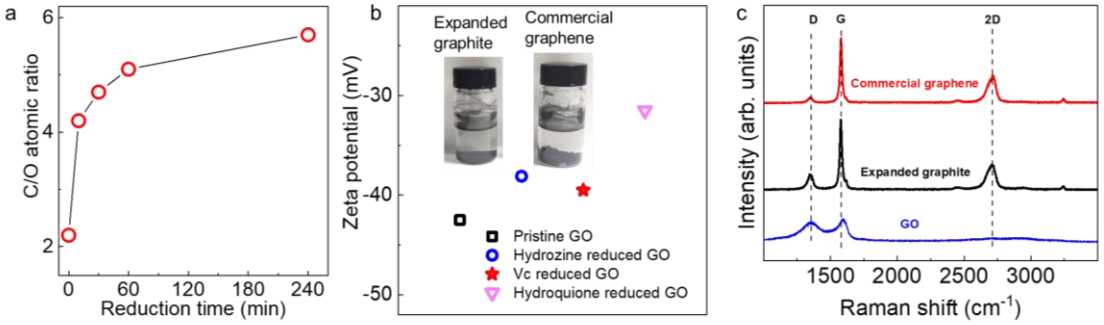

Supplementary Fig. 12| Evaluation of different graphene-based adsorbents. (a) C/O atomic ratio of rGO with different reduction time using ascorbic acid. (b) Zeta potentials of GO and different rGO suspensions. Insets: photographs show that no stable dispersion of nanosheets of graphene or expanded graphite could be achieved in water. (c) Raman spectra of the GO, expanded graphite and commercial graphene.

Supplementary Note 6. Extraction of gold from seawater

As an initial test for the selectivity of rGO, we measured its uptake of metals from an aqueous solution containing 10 ppm of each Au, Cu, Ni and Pt, using salts $KAuCl_4$, $CuCl_2$, $CuSO_4$, $Cu(NO_3)_2$, $NiCl_2$ and K_2PtCl_4. As shown in Supplementary Fig. 13a, rGO allowed recovery of ~99% of Au from the mixture whereas only ~5% Cu, 1.4% Pt and 1% Ni were adsorbed on rGO. Similar % values were also found using 10 ppm solutions of the individual salts rather than their mixture (inset of Supplementary Fig. 13a), as expected for non-interacting hydrated ions. Not only the metal

cationic ions, the above experiments also suggested, there was no noticeable influence of the co-existing anionic ions including Cl^-, NO^{3-}, SO_4^{2-}, $[PtCl_4]^-$ on the gold extraction performance. This was probably because there was no specific interaction for these anions with graphene or oxidized regions. This behaviour suggests that graphene exhibits preferential affinity to gold as compared to the co-existing ions (Supplementary Fig. 13b).

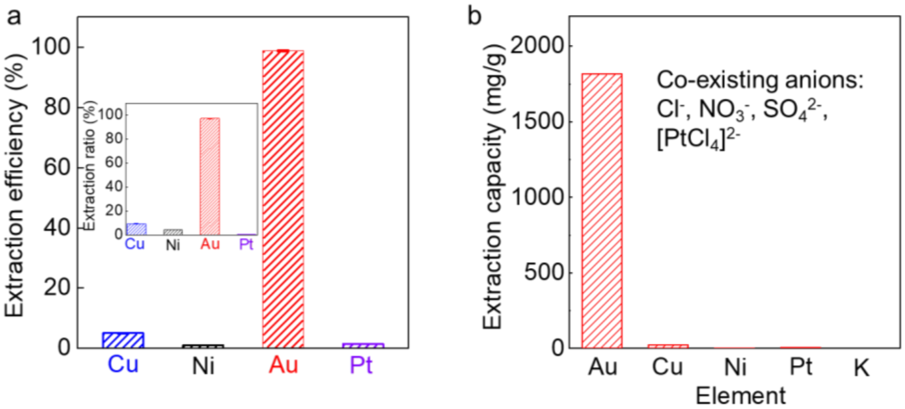

Supplementary Fig. 13| Extraction selectivity of rGO. (a) Extraction efficiency for an equal-part mixture of $[AuCl_4]^-$, Cu^{2+}, Ni^{2+} and $[PtCl_4]^{2-}$ ions. Inset: Same for aqueous solutions containing individual metal ions. (b) The influence of the co-existing cations and anions for gold extraction capacity using rGO. All the error bars in this figure represent the standard deviation.

To demonstrate the effectiveness of rGO for gold extraction from solutions containing many different salts, we prepared a simulated seawater solution containing sodium, magnesium, calcium and potassium ions in concentrations typical for oceans. Then 100 ppb of gold ions were added to this solution. First, we carried out the standard extraction protocol from the simulated seawater using pH ≈ 4. The rGO's uptake of gold was found > 99% but with significant presence of Na, Ca and K (Supplementary Fig. 14). After adding an extra hour at pH ≈ 1 (that is, using protocol 2), the gold uptake increased even further with no noticeable presence of Na and Ca. The remaining K ions (~4% uptake after protocol 2) could be removed by washing away the potassium salts adsorbed on rGO by simply rinsing rGO in water. This shows that the proposed extraction protocols can be adapted to many different situations to achieve a highly selective extraction of Au (Supplementary Fig. 14).

In further experiments, 0.3 mg of rGO was added to 200 mL of the simulated seawater spiked with $KAuCl_4$ to achieve absolutely minute concentrations of gold ions (10 ppt). After 2 days of extraction, the rGO was collected by centrifugation and drop-casted on a Si wafer for XPS. Similar to the ppt level of gold in pure water, we observed varied gold contents (0.01-0.11 at%) at different

areas, which translated into an extraction capacity even higher than the theoretical capacity (calculated based on 100 % extraction efficiency), such high gold content in rGO measured by XPS allowed us to estimate a complete extraction to gold solution at ppt level.

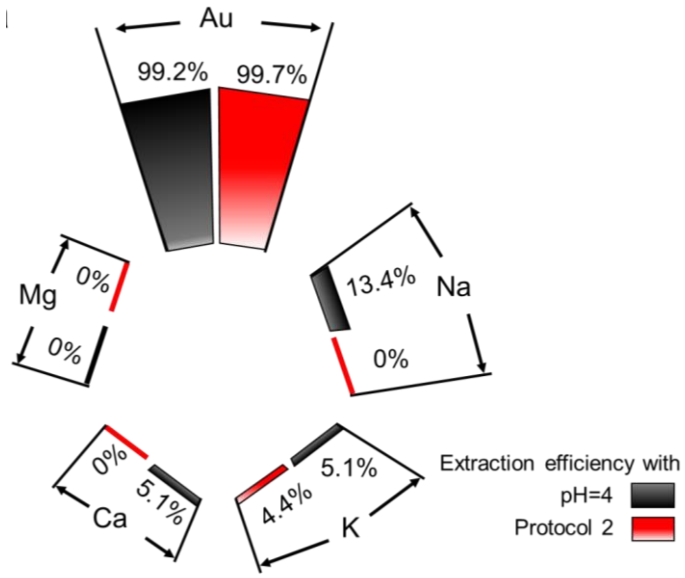

Supplementary Fig. 14| Gold extraction from simulated seawater. Highly selective extraction from the seawater with added 100 ppb of gold. Comparison of efficiencies for different extraction protocols (colour coded).

Supplementary Note 7. Gold extraction from e-waste

In our demonstration of e-waste recycling in Fig. 3b, a CPU leachate was diluted to emphasize the superior performance of rGO for extracting even trace amounts of gold and make sure all the gold ions were discharged in the leachate by repeated washing. In real conditions, CPU leachates would contain gold in concentrations from a few to tens of ppm[1,2]. Therefore, we also performed Au extraction from real-world CPU leachates. Specifically, our CPU leachate contained 2.65 ppm Au, 106 ppb tin (Sn), 13 ppb chromium (Cr), 8.5 ppm aluminium (Al), 9.6 ppm Ca, 1 ppb lead (Pb), 54 ppb Ni, 242 ppm Cu, 170 ppb Mg, 68 ppb iron(Fe), 200 ppb zinc (Zn), 70 ppb strontium (Sr), 8 ppb arsenic (As), 620 ppb barium (Ba), and 13 ppb manganese (Mn). Supplementary Fig. 15 shows that rGO remained very effective with 99% of gold being extracted. Using the pH=4 protocol, rGO also adsorbed approximately 97% Sn, 87% Cr, 62% Al, 39% Pb, 98% As and 2.4% Cu with no discernible adsorption of the other metals present in the leachate. In contrast, if protocol 2 was used, >99% of gold was extracted from the CPU leachate with neither of the 14 coexisting ions being present on rGO (Supplementary Fig. 15). The ability to achieve such exclusive gold extraction

in the presence of many other ions shows an unambiguous potential of the proposed technology for recycling of e-waste.

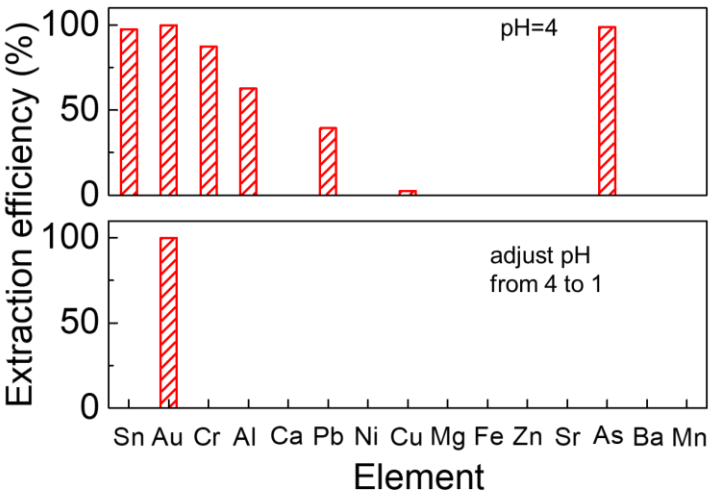

Supplementary Fig. 15| rGO performance for gold extraction from real-world CPU leachates. Top and bottom panels are for different extraction protocols.

Supplementary Note 8. Continuous gold extraction using rGO membranes

8.1 Optimal membrane thickness and characterization of extracted gold

To find an optimal thickness of rGO membranes for continuous gold extraction, we measured the solution permeance and extraction efficiencies for membranes having thicknesses of ~ 0.2, 0.8 and 2 μm. 20 ml of a 100 ppm Au solution was filtered through them. For convenience, to measure the relatively high gold concentrations in the feed and filtrate solutions, we used UV-Vis spectroscopy. It showed an absorption peak at ~290 nm, which intensity changed linearly with increasing the gold concentration (bottom inset of Supplementary Fig. 16a). This peak was then used in real time to determine gold concentrations in the filtrate and to calculate the uptake. Supplementary Fig. 16a shows that permeance of the membranes decreased with increasing their thickness, as a higher flow resistance is obviously expected for thicker membranes. On the other hand, the extraction efficiency increased with increasing the membrane thickness, which is also expected because thicker membranes get more rGO nanosheets involved in the extraction process. The thickness-dependent trade-off between efficiency and permeance suggests that the continuous extraction process can be adjusted to reach desirable performance by changing the membrane's thickness. Note that, in the described experiments, we did not try to reach highest extraction efficiencies because of rather high

Au concentrations and the limited amount of rGO such that the membranes were unable to adsorb all gold present in the tested solution during its single filtration pass.

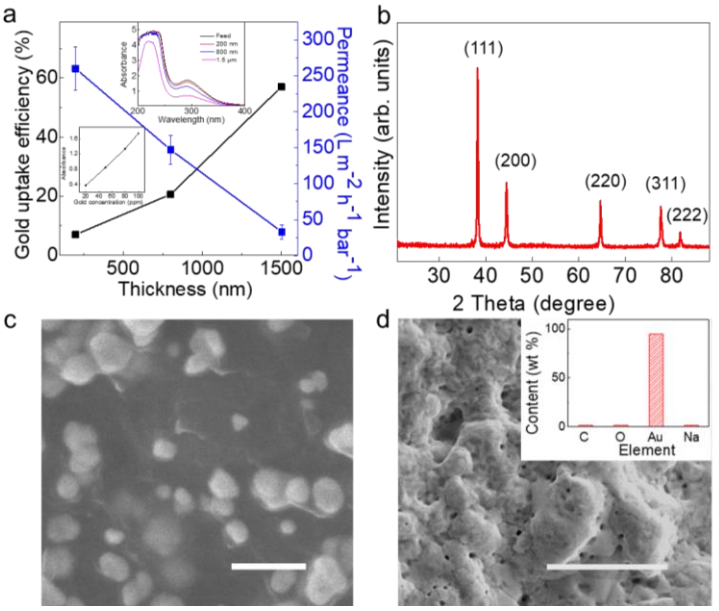

Supplementary Fig. 16| Performance of rGO membranes and analysis of the extracted gold. (**a**) Permeance and gold uptake for membranes with various thickness; 20 mL of a $KAuCl_4$ solution was filtered through. Left inset: intensity of the $[AuCl_4]^-$ ion absorption peak at ~290 nm for different Au ion concentrations. The upper inset shows UV-Vis absorption spectra of the feed solution and the filtrate. (**b**) XRD pattern of an rGO membrane (~800 nm thick) after filtration of 6.6 L of a 100 ppb $[AuCl_4]^-$ solution. (**c**) SEM micrograph of the membrane's surface after the extraction. Scale bar, 200 nm. (**d**) SEM image of the particulates left after burning off the rGO membrane. Scale bar, 5 μm. Inset: EDS analysis of the particulate. All the error bars in this figure represent the standard deviation.

Next, we filtered a 6.6 L of a dilute $[AuCl_4]^-$ solution (100 ppb) through an rGO membrane (3 cm^2; 800 nm thick). After the filtration the membrane was exfoliated from its polymer support and studied by SEM. As shown in Supplementary Fig. 16, many nanoparticles were found on top and inside the rGO membrane. XRD confirmed that they were metallic gold. Burning off the rGO membrane resulted in a deposit containing 95.2 wt% Au, 1.7 wt% Na, 1.67 wt% C, and 1.43 wt% O, as measured by EDS (Supplementary Fig. 16d). The purity of the resulting gold is calculated to be 23 carats. Based on the TG analysis of pristine rGO (Supplementary Fig. 4c), the small amounts of detected carbon, oxygen and sodium come not from our extraction process but were probably due to the residual ash of rGO and contamination of the gold surface during its SEM analysis.

8.2 <u>Continuous extraction from CPU leachates</u>

In this experiment, we used an rGO membrane to filter a diluted CPU leachate containing 100 ppb of Au. This was designed to check the effectiveness of the proposed continuous extraction rather than to deal with real Au leachates having typically much higher concentrations of Au (see above). Also, we did not carry out this particular experiment in a single filtration step as in Fig. 4. Instead, seven cycles of filtration were used until the 50% permeance was reached (Supplementary Fig. 17). After each cycle, the membrane was soaked in concentrated HCl at pH =1. Supplementary Fig. 15 shows the permeance and extraction efficiency over those 7 cycles (140 mL in total was filtrated through). Similar to the case of continuous extraction from solutions containing gold ions only (Fig. 4b), we found that the permeance decreased with each extraction cycle because of the blocking of rGO with metallic gold. After each filtration cycle, we implemented soaking in HCl to desorb coexisting ions from rGO (Supplementary Fig. 17b). However, this desorption required long time (typically 48 hours) because, unlike rGO colloids, rGO membranes consisted of narrow nanochannels, and it took time for HCl to diffuse and remove adsorbed coexisting ions. Note that the long soaking step could in principle be avoided or shortened by reducing the leachate's pH (soaking and filtration occur simultaneously) or by adding sonication to enhance the diffusion of HCl. Nevertheless, even without further optimization, our results clearly show a possibility of using the proposed rGO technology for continuous gold extraction with exceptional performance.

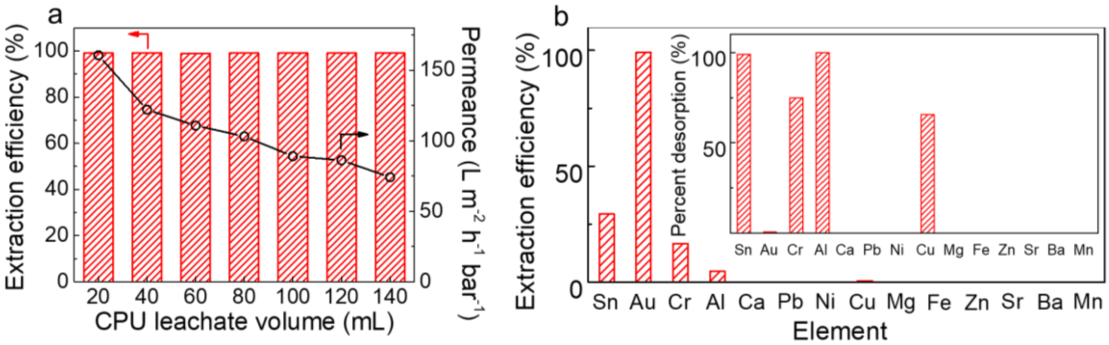

Supplementary Fig. 17| Continuous and highly selective extraction of Au from CPU leachates. (**a**) Changes in extraction efficiency and water permeance through an 800 nm thick rGO membrane during (quasi)continuous filtration. (**b**) Extraction efficiency for different chemical elements present in CPU leachates. Inset: Percentage of coexisting ions removed from the rGO membrane by soaking it in an HCl solution for 2 days.

8.3 Recovery of copper from e-waste

After extracting gold from our CPU leachates by continuous filtration, the filtrate contained a significant amount of copper and other coexisting elements. We demonstrate that this copper can

also be recovered in a simple process. By adding iron particulates to the leachate remaining after Au extraction, copper inside the solution was reduced (galvanic replacement) and precipitated. The precipitate was collected and analysed by SEM and EDS, which revealed that it contained > 95% of copper (Supplementary Fig. 18).

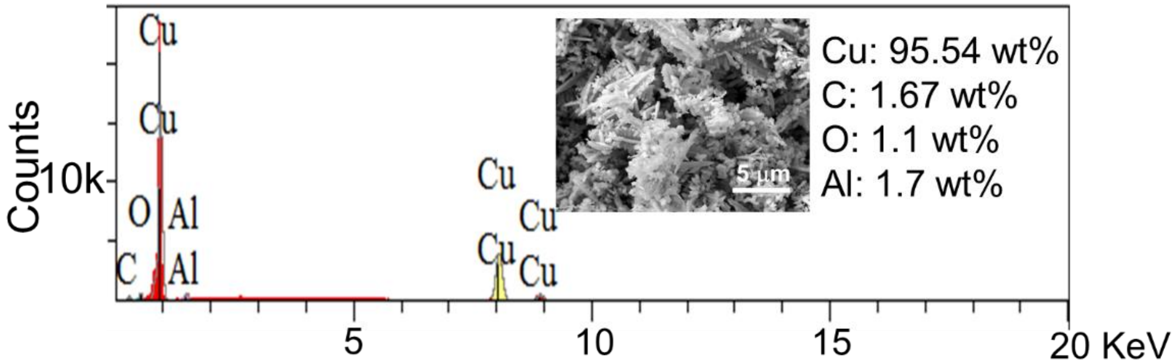

Supplementary Fig. 18| EDS analysis of the copper recovered from e-waste. Inset: SEM micrograph of the recovered copper and its elemental composition.

Supplementary References

1. D. T. Sun, N. Gasilova, S. Yang, E. Oveisi, W. L. Queen, Rapid, selective extraction of trace amounts of gold from complex water mixtures with a metal-organic framework (MOF)/polymer composite. *J. Am. Chem. Soc.* **140**, 16697-16703 (2018).
2. Y. Hong *et al.*, Precious metal recovery from electronic waste by a porous porphyrin polymer. *Proc. Natl. Acad. Sci. U.S.A.* **117**, 16174 (2020).
3. A. D. Dwivedi, S. P. Dubey, S. Hokkanen, R. N. Fallah, M. Sillanpää, Recovery of gold from aqueous solutions by taurine modified cellulose: an adsorptive–reduction pathway. *Chem. Eng. J.* **255**, 97-106 (2014).
4. P. Chen, Y. Liang, B. Yang, F. Jia, S. Song, In situ reduction of Au(I) for efficient recovery of gold from thiosulfate solution by the 3D MoS_2/chitosan aerogel. *ACS Sustain. Chem. Eng.* **8**, 3673-3680 (2020).
5. T. S. Nguyen, Y. Hong, N. A. Dogan, C. T. Yavuz, Gold recovery from e-waste by porous porphyrin–phenazine network polymers. *Chem. Mater.* **32**, 5343-5349 (2020).
6. C. Wang *et al.*, Highly selective recovery of Au(III) from wastewater by thioctic acid modified Zr-MOF: experiment and DFT calculation. *Chem. Eng. J.* **380**, 122511 (2020).
7. L. Liu *et al.*, Preparation and characterization of chitosan/graphene oxide composites for the adsorption of Au(III) and Pd(II). *Talanta* **93**, 350-357 (2012).
8. M. Mon *et al.*, Selective gold recovery and catalysis in a highly flexible methionine-decorated metal–organic framework. *J. Am. Chem. Soc.* **138**, 7864-7867 (2016).
9. S. Lin *et al.*, Effective adsorption of Pd(ii), Pt(iv) and Au(iii) by Zr(iv)-based metal–organic frameworks from strongly acidic solutions. *J. Mater. Chem. A* **5**, 13557-13564 (2017).
10. C. Wu *et al.*, Specific recovery and in situ reduction of precious metals from waste to create

MOF composites with immobilized nanoclusters. *Ind. Eng. Chem. Res.* **56**, 13975-13982 (2017).

11. K. F. Lam, C. M. Fong, K. L. Yeung, G. McKay, Selective adsorption of gold from complex mixtures using mesoporous adsorbents. *Chem. Eng. J.* **145**, 185-195 (2008).
12. R. Chand *et al.*, Selective adsorption of precious metals from hydrochloric acid solutions using porous carbon prepared from barley straw and rice husk. *Miner. Eng.* **22**, 1277-1282 (2009).
13. O. F. Odio, L. Lartundo-Rojas, P. Santiago-Jacinto, R. Martínez, E. Reguera, Sorption of gold by naked and thiol-capped magnetite nanoparticles: an XPS approach. *J. Phys. Chem. C* **118**, 2776-2791 (2014).
14. K. Fujiwara, A. Ramesh, T. Maki, H. Hasegawa, K. Ueda, Adsorption of platinum (IV), palladium (II) and gold (III) from aqueous solutions onto l-lysine modified crosslinked chitosan resin. *J. Hazard. Mater.* **146**, 39-50 (2007).
15. D. Parajuli *et al.*, Selective recovery of gold by novel lignin-based adsorption gels. *Ind. Eng. Chem. Res.* **45**, 8-14 (2006).
16. J. Wang, J. Li, J. Wei, Adsorption characteristics of noble metal ions onto modified straw bearing amine and thiol groups. *J. Mater. Chem. A* **3**, 18163-18170 (2015).
17 F. Liu, S. You, Z. Wang, Y. Liu, Redox-active nanohybrid filter for selective recovery of gold from water. *ACS EST Engg.* **1**, 1342-1350 (2021).
18 T. Ma et al., Efficient gold recovery from e-waste via a chelate-containing porous aromatic framework. *ACS Appl. Mater. Interfaces* **12**, 30474-30482 (2020).
19. F. Yang et al., Rapid capture of trace precious metals by amyloid-like protein membrane with high adsorption capacity and selectivity. *J. Mater. Chem. A* **8**, 3438-3449 (2020).
20. W. Zhan, Y. Yuan, B. Yang, F. Jia, S. Song, Construction of MoS_2 nano-heterojunction via ZnS doping for enhancing in-situ photocatalytic reduction of gold thiosulfate complex. *Chem. Eng. J.* **394**, 124866 (2020).
21. B. Feng et al., Highly efficient and selective recovery of Au(III) from a complex system by molybdenum disulfide nanoflakes. *Chem. Eng. J.* **350**, 692-702 (2018).
22. J. M. Wasikiewicz, N. Nagasawa, M. Tamada, H. Mitomo, F. Yoshii, Adsorption of metal ions by carboxymethylchitin and carboxymethylchitosan hydrogels. *Nucl. Instrum. Meth. B* **236**, 617-623 (2005).
23. W. S. Wan Ngah, K. H. Liang, Adsorption of gold (III) ions onto chitosan and N-carboxymethyl chitosan: equilibrium studies. *Ind. Eng. Chem. Res.* **38**, 1411-1414 (1999).
24. M. J. Fernández-Merino *et al.*, Vitamin C is an ideal substitute for hydrazine in the reduction of graphene oxide suspensions. *J. Phys. Chem. C* **114**, 6426-6432 (2010).
25. Montero, M. A., Gennero de Chialvo, M. R. & Chialvo, A. C, Preparation of gold nanoparticles supported on glassy carbon by direct spray pyrolysis. *J. Mater. Chem.* **19**, 3276-3780 (2009).
26. L. G. Cançado *et al.*, Quantifying defects in graphene via raman spectroscopy at different excitation energies. *Nano Lett.* **11**, 3190-3196 (2011).
27. F. Güneş et al., Layer-by-layer doping of few-layer graphene film. *ACS Nano* **4**, 4595-4600 (2010).
28. J. Li, C. Y. Liu, Y. Liu, Au/graphene hydrogel: synthesis, characterization and its use for catalytic reduction of 4-nitrophenol. *J. Mater. Chem.* **22**, 8426-8430 (2012).

29. Geim, A. K. & Novoselov, K. S, The rise of graphene. *Nat. Mater.* **6**, 183-191 (2007).
30. Sun, P. Z. *et al.*, Limits on gas impermeability of graphene. *Nature* **579**, 229-232 (2020).
31. G. Kresse and J. Furthmuller, Efficiency of ab-initio total energy calculations for metals and semiconductors using a plane-wave basis set. *Comput. Mater. Sci.* **6**, 15 (1996).
32. G. Kresse and J. Furthmuller, Efficient iterative schemes for ab initio total-energy calculations using a plane-wave basis set. *Phys. Rev. B* **54**, 11169 (1996).
33. P. Hohenberg and W. Kohn, Inhomogeneous electron gas. *Phys. Rev.* **136**, B864 (1964).
34. W. Kohn and L. J. Sham, Self-consistent equations including exchange and correlation effects. *Phys. Rev.* **140**, A1133 (1965).
35. J. P. Perdew, K. Burke and M. Ernzerhof, Generalized gradient approximation made simple. *Phys. Rev. Lett.* **77**, 3865 (1996).
36. S. Grimme, J. Antony, S. Ehrlich and H. Krieg, A consistent and accurate ab initio parametrization of density functional dispersion correction (DFT-D) for the 94 elements H-Pu. *J. Chem. Phys.* **132**, 154104 (2010).
37. M. Chagas da Silva et al., Self-consistent potential correction for charged periodic systems. *Phys. Rev. Lett.* **126**, 076401 (2021).
38. S. Pei, H.-M. Cheng, The reduction of graphene oxide. *Carbon* **50**, 3210-3228 (2012).
39. S. Honary, F. Zahir, Effect of zeta potential on the properties of nano-drug delivery systems - a review (Part 1). *Trop. J. Pharm. Res.* **12**, 255-264 (2013).
