# Supplementary material for: Highly efficient and selective extraction of gold by reduced graphene oxide
Source: Nat Commun. 2022 Aug 2;13:4472. doi: 10.1038/s41467-022-32204-4 (PMC9345893; doi:10.1038/s41467-022-32204-4)
Supplement: Supplementary file 3 — Description of Additional Supplementary Files [file 41467_2022_32204_MOESM3_ESM.pdf]

Description of Additional Supplementary Files

File Name: Supplementary Movie 1
Description: The rGO membrane based extraction to 1 ppm gold solution
